# *In vivo* longitudinal study of rodent skeletal muscle atrophy using ultrasonography

**DOI:** 10.1038/srep20061

**Published:** 2016-02-01

**Authors:** Antonietta Mele, Adriano Fonzino, Francesco Rana, Giulia Maria Camerino, Michela De Bellis, Elena Conte, Arcangela Giustino, Diana Conte Camerino, Jean-François Desaphy

**Affiliations:** 1 Section of Pharmacology, Department of Pharmacy & Drug Sciences, University of Bari Aldo Moro, Bari, 70125 Italy; 2 Department of Biomedical Sciences & Human Oncology, Polyclinic Biological Research Institute, University of Bari Aldo Moro, P.zza Giulio Cesare 11, Bari, 70124 Italy

## Abstract

Muscle atrophy is a widespread ill condition occurring in many diseases, which can reduce quality of life and increase morbidity and mortality. We developed a new method using non-invasive ultrasonography to measure soleus and gastrocnemius lateralis muscle atrophy in the hindlimb-unloaded rat, a well-accepted model of muscle disuse. Soleus and gastrocnemius volumes were calculated using the conventional truncated-cone method and a newly-designed sinusoidal method. For Soleus muscle, the ultrasonographic volume determined *in vivo* with either method was linearly correlated to the volume determined *ex-vivo* from excised muscles as muscle weight-to-density ratio. For both soleus and gastrocnemius muscles, a strong linear correlation was obtained between the ultrasonographic volume and the muscle fiber cross-sectional area determined *ex-vivo* on muscle cryosections. Thus ultrasonography allowed the longitudinal *in vivo* evaluation of muscle atrophy progression during hindlimb unloading. This study validates ultrasonography as a powerful method for the evaluation of rodent muscle atrophy *in vivo*, which would prove useful in disease models and therapeutic trials.

Muscle atrophy is a widespread ill condition occurring during inactivity, aging, and various diseases, including neuromuscular disorders, cancer, bacterial and viral infections, chronic lung and kidney diseases, diabetes, and drug side effects^1^,^2^. The loss of muscle mass and function can reduce quality of life and increase morbidity and mortality. While exercise is today the only recognized counteracting measure to slow atrophy, a number of studies in the last decade have shed light on the underlying molecular mechanisms, paving the way for drug development. This later will require preclinical models and associated powerful techniques to evaluate trial outcomes. To date the measure of muscle atrophy in animal disease models usually requires animal sacrifice in order to weigh excised muscles and perform histological and biochemical studies. This approach is invasive and expensive involving the use of a large number of animals to obtain significant results. Therefore realizing a new, non invasive method allowing longitudinal *in vivo* evaluation of muscle atrophy would become an invaluable tool.

The ultrasonography is a non invasive diagnostic imaging technique based on the application of ultrasounds, which is widely used for various medical applications, including the quantification of structural and functional changes in skeletal muscles^3^,^4^. In the recent years, the technique has been adapted to the preclinical setting, owing to the development of equipment able to work at high frequencies, from 40 to 100 MHz, and therefore suitable for high-resolution ultrasound evaluations on small animals such as rodents^5^. While ultrasonography has been mostly used for tumor and cardiac investigations^6^,^7^,^8^,^9^, there are currently only a few reports relative to its use for the evaluation of skeletal muscle structural and functional parameters in rodents. The aim of this study was to develop a non-invasive method to evaluate *in vivo* the volume variation of hindlimb muscle of rats, as a measure of skeletal muscle atrophy, using ultrasonography. To achieve this goal, we performed a longitudinal ultrasonographic study of rat soleus (Sol) and gastrocnemius lateralis (Gas) muscle volume variation during a 14-days hindlimb-unloading (HU) period, which is a widely acknowledged model of disuse-induced muscle atrophy^10^,^11^.

## Results and Discussion

**Figure 1:**
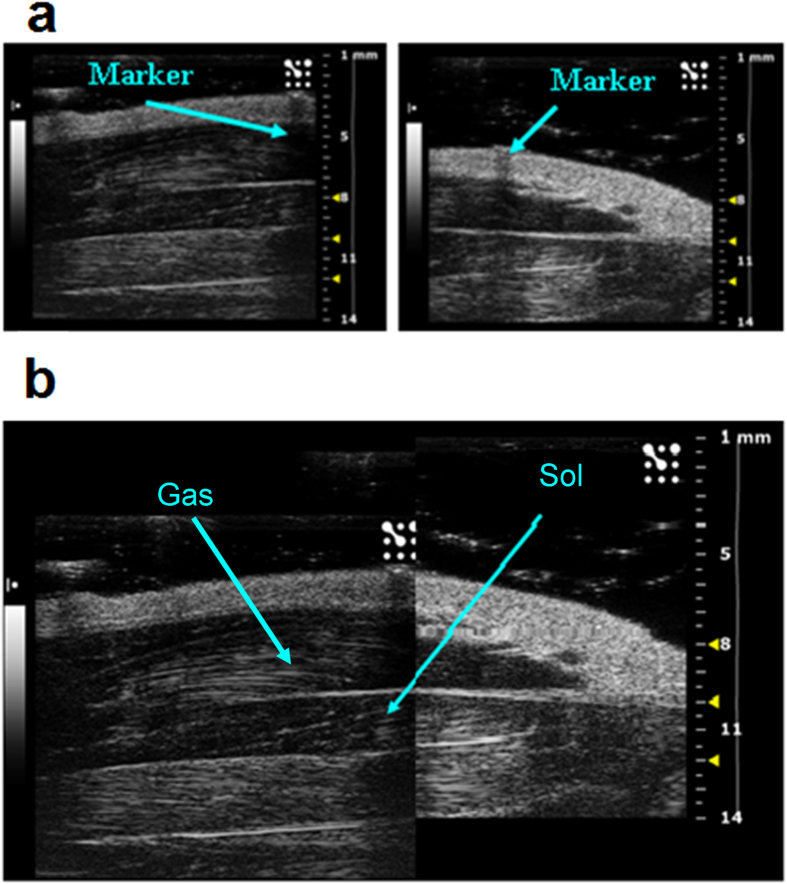
Acoustic window used to visualize whole soleus (Sol) muscle. (**a**) Sample images acquired from proximal position (left) and distal position (right). Above the skin, it is possible to visualize the hyperechogenic marker used to separate the proximal portion from the distal one. (**b**) Graphic reconstruction of the whole hindlimb, from ankle (left side) to knee (right side). The images was obtained graphically integrating the distal and the proximal images. The soleus and gastrocnemius lateralis muscles are indicated by arrows.

**Figure 2:**
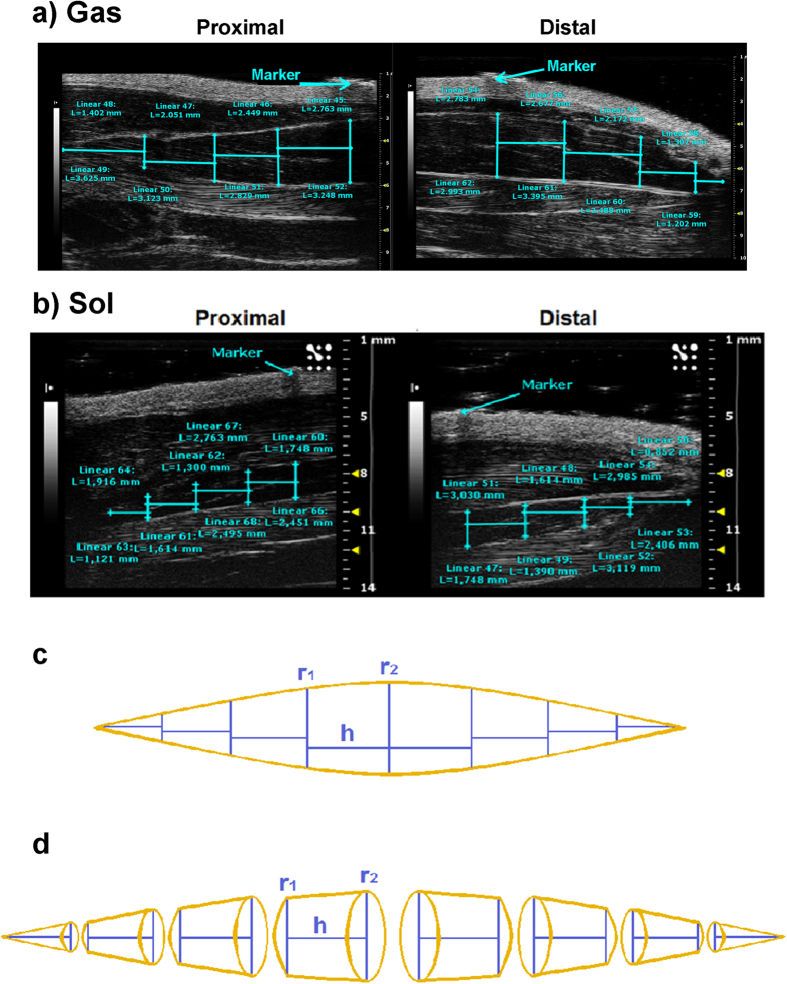
Representative ultrasonographic images of truncated cone calculation method of gastrocnemius and soleus muscle volumes. (**a**) Proximal and distal acquisitions of the Gas muscle. In cyan are visualized the measurement lines used to calculate the Gas muscle volume using the truncated-cone formula. Images were acquired by B-mode. Probe: MS550D (VisualSonics, Fujifilm, Toronto). (**b**) Proximal and distal acquisitions of the Sol muscle. **(c)**. Graphical representation of the soleus muscle as it appears in the acoustic window, showing the minor radius (r_1_), the major radius (r_2_) and the height (h) of each truncated cone. (**d**) Three-dimensional representation of the truncated cones forming the whole soleus muscle volume.

**Figure 3:**
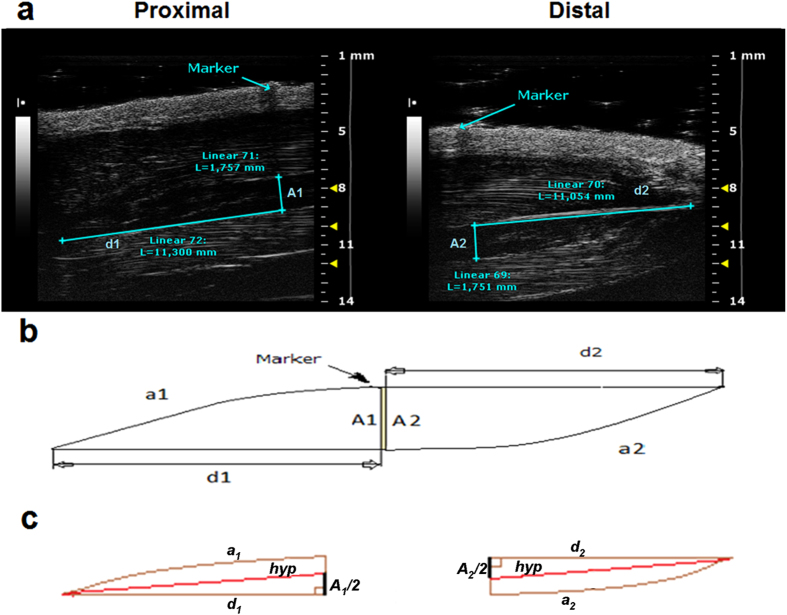
Representative ultrasonographic images of the novel sinusoidal calculation method of soleus muscle volume. (**a**) Proximal and distal acquisitions of the Sol muscle. In cyan are visualized the measurement lines used to calculate the muscle volume using the sinusoidal method. Images were acquired by B-mode. Probe: MS550D (VisualSonics, Fujifilm, Toronto) (**b**) Graphical representation of the soleus muscle as it appears in the acoustic window, showing the major catheti, d_1_ and d_2_, the sinusoidal arches, a_1_ and a_2_, and the greater thickness, A_1_ and A_2_. (**c**) Geometric shape of the proximal and distal soleus muscle, showing right triangles with major catheti d_1_ or d_2_, minor catheti, A_1_/2 or A_2_/2, and hypotenuses hyp.

**Figure 4:**
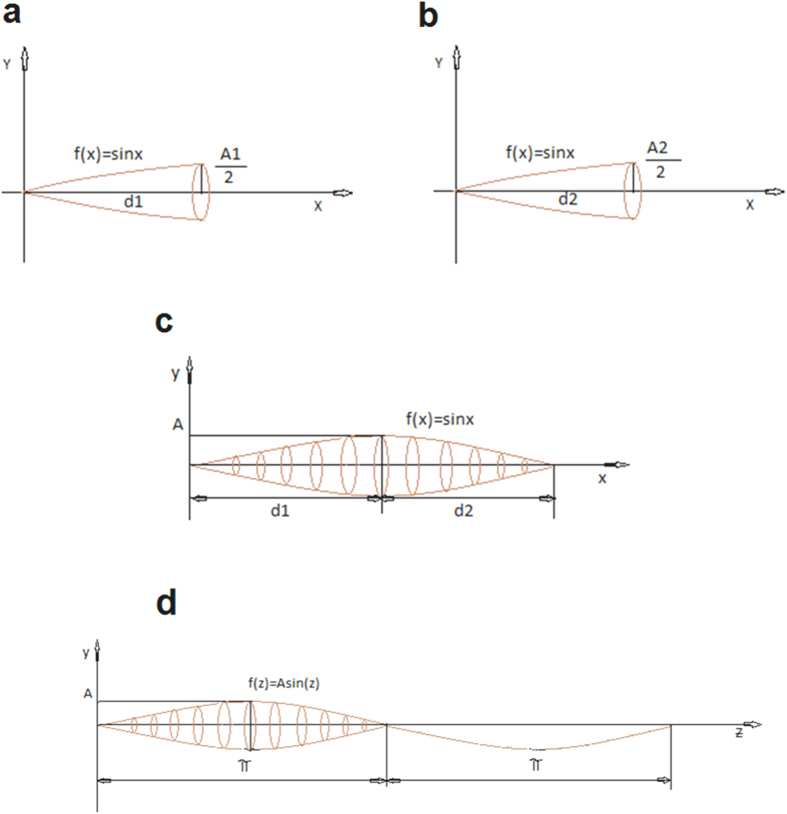
Representation of the solid of rotation of the sine function. (**a**) The proximal part of the muscle is approximated as a solid of rotation around the cathetus d_1_. (**b**) The distal part of the muscle is approximated as a solid of rotation around the cathetus d_2_. (**c**) Whole solid of rotation resulting by the assembly of the proximal and distal solid rotation. (**d**) Solid of rotation of the sine function in which the x variable has been replaced with the new z variable.

**Figure 5:**
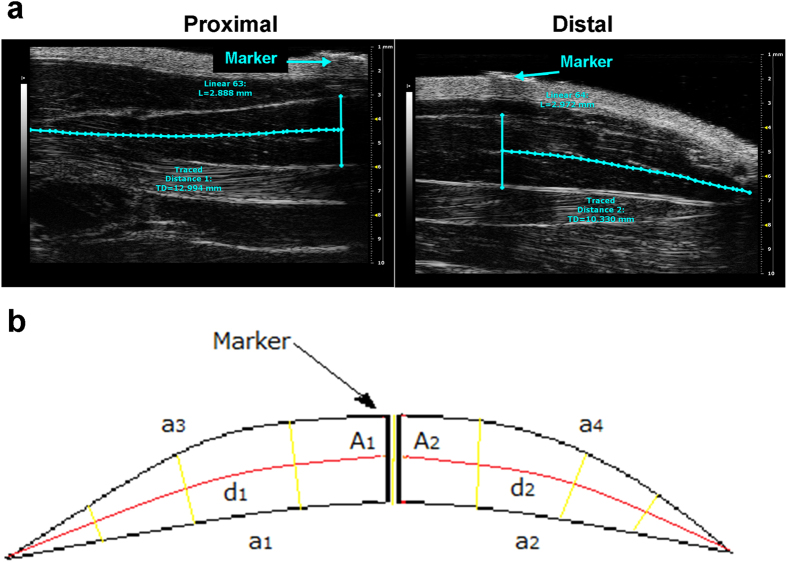
Representative ultrasonographic images of the novel sinusoidal calculation method of gastrocnemius muscle volume. (**a**) Proximal and distal acquisitions of the Gas muscle. In cyan are visualized the measurement lines used to calculate the muscle volume using the sinusoidal method. Images was acquired by B-mode. Probe: MS550D (VisualSonics, Fujifilm, Toronto). (**b**) Graphical representation of the gas muscle as it appears in the acoustic window, showing the symmetry lines, d_1_ and d_2_, the sinusoidal arches, a_1_ and a_2_, and the greater thickness, A_1_ and A_2_.

Ultrasound B-Mode acquisitions of Sol and Gas muscle images were performed at day 0 (D0), 7 (D7) and 14 (D14) in control (CTRL) and hindlimb unloaded (HU) adult rats. The anaesthetized rat was placed in the ventral decubitus position, and the 40-MHz probe was fixed parallel to the hindlimb (Supplementary Fig. 1). In this condition, it was possible to visualize the whole length of the Sol and Gas muscles tendon-to-tendon by shifting the probe along the longitudinal axis. For image analysis, the Sol and Gas muscles were virtually divided in distal and proximal parts with approximately similar lengths (Fig. 1a,b). The Sol and Gas volumes were calculated by using both the conventional truncated-cone method, which is currently used for the assessment of human muscle volume^12^, and a newly-designed sinusoidal method (*see* methods). The first method consists in summing the calculated volumes of designed truncated cones covering the whole muscle image (Fig. 2). The new sinusoidal method considers the fusiform profile of the spindle-shaped Sol and Gas muscles resembling a sinusoidal function, and the Sol and Gas volumes are approximated by the rotation around the tendon-to-tendon axis of a sine function (Figs 3, 4, 5).

**Figure 6:**
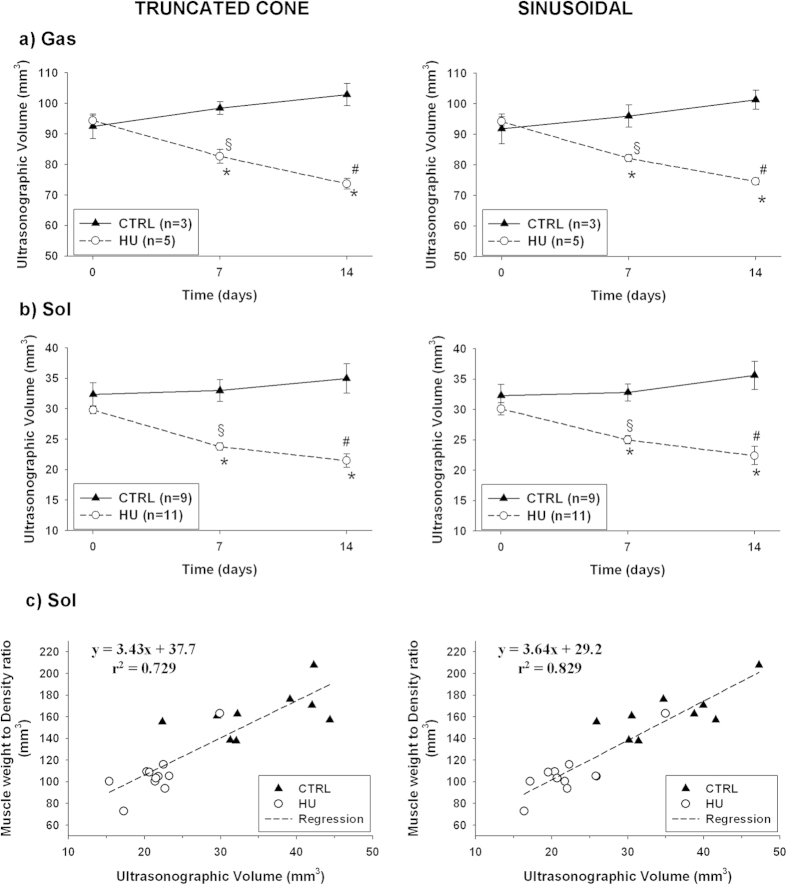
Comparison between the truncated-cone (left column) and sinusoidal (right column) methods for calculation of Sol and Gas muscle volumes from ultrasonographic acquisitions. (**a**) Time-course of ultrasonographic Gas volumes in control (CTRL, n = 3) and hindlimb-unloaded rats (HU, n = 5). Data are expressed as mean ± SEM. Paired Student’s t-test indicates at least p < 0.02 versus HU-D0 (*). Unpaired Student’s t-test indicates at least p < 0.005 versus CTRL-D7 (§) and p < 0.0002 versus CTRL-D14 (#). (**b**) Time-course of ultrasonographic Sol volumes in control (CTRL, n = 9) and hindlimb-unloaded rats (HU, n = 11). Data are expressed as mean ± SEM. Paired Student’s t-test indicates at least p < 0.001 versus HU-D0 (*). Unpaired Student’s t-test indicates at least p < 0.0001 versus CTRL-D7 (§) and p < 0.0002 versus CTRL-D14 (#). (**c**) Linear correlation between ultrasonographic Sol volumes and Sol muscle weight-to-density ratio in CTRL (n = 9) and HU (n = 11) rats.

The results showed that, independently on the calculation method, no significant changes of Sol and Gas muscle volumes occur in CTRL rats between D0, D7 and D14 (Fig. 6a,b). Within the HU group, a significant reduction of Sol and Gas muscle volumes was observed at D7 and D14 with respect to D0, both with the truncated-cone and sinusoidal methods (at least p < 0.01 with paired Student’s *t*-test) (Fig. 6a,b). Note that no significant difference was found between the right and left limbs of HU rats (not shown), and muscle volumes of both limbs were averaged to obtain a single value for each animal. Regarding Gas muscle, the percentage reduction of Sol muscle volume at D7 and D14 was −12.4 ± 2.2% and −21.9 ± 1.7%, respectively, with the truncated cone method, and −12.7 ± 1.3% and −20.8 ± 1.2% with the sinusoidal method. Consistently, the Gas muscle volume of HU rats (n = 5) was significantly reduced with respect to that of CTRL animals (n = 3) at D7 (−16.0 ± 2.2% and −14.4 ± 2.9%, truncated-cone and sinusoidal methods, p < 0.005 with unpaired Student’s *t*-test) and D14 (−28.4 ± 2.2% and −26.4 ± 2.0%, p < 0.0002) (Fig. 6a). Compared to D0, the percentage reduction of Sol muscle volume at D7 and D14 was −20.1 ± 1.9% and −27.9 ± 3.5%, respectively, with the truncated cone method, and −16.9 ± 2.6% and −25.6 ± 4.6% with the sinusoidal method. With respect to that of CTRL animals (n = 9), the Sol muscle volume of HU rats (n = 11) was significantly reduced at D7 (−27.9 ± 3.6% and −23.8 ± 2.9%, truncated-cone and sinusoidal methods, p < 0.0001 with unpaired Student’s *t*-test) and D14 (−38.6 ± 3.8% and −37.1 ± 4.2%, p < 0.0002) (Fig. 6b). These values are in line with the percentage reductions observed in previous studies, in which Sol or Gas muscle atrophy was evaluated *ex-vivo* as a reduction of muscle weight^13^,^14^,^15^,^16^,^17^,^18^,^19^.

At D14, a linear correlation (y = *a*x + *b*) between the ultrasonographic volumes and the weight-to-density ratio of Sol muscles excised from the same animal was observed, showing a coefficient of determination r^2^ of 0.73 and 0.83 with the truncated-cone or the sinusoidal methods, respectively (Fig. 6c). Despite the fact that we can quantify the Sol muscle volume variations, we cannot directly measure the actual absolute Sol muscle volume using ultrasonography. Such a difference is at least partially due the actual ellipse-like cross section of the Sol muscle, which is underestimated by the circular cross section of the truncated cones or the sinusoidal spindle. Nevertheless, the linear correlation provides us with the possibility to satisfactorily approximate the actual soleus volume from that measured with ultrasonographic technique (Fig. 6c). No such satisfactory correlation was obtained for Gas muscle (r^2^ = ∼0.5, not shown), due to technical limitation in the excision of Gas muscles resulting in highly variable Gas muscle weights.

**Figure 7:**
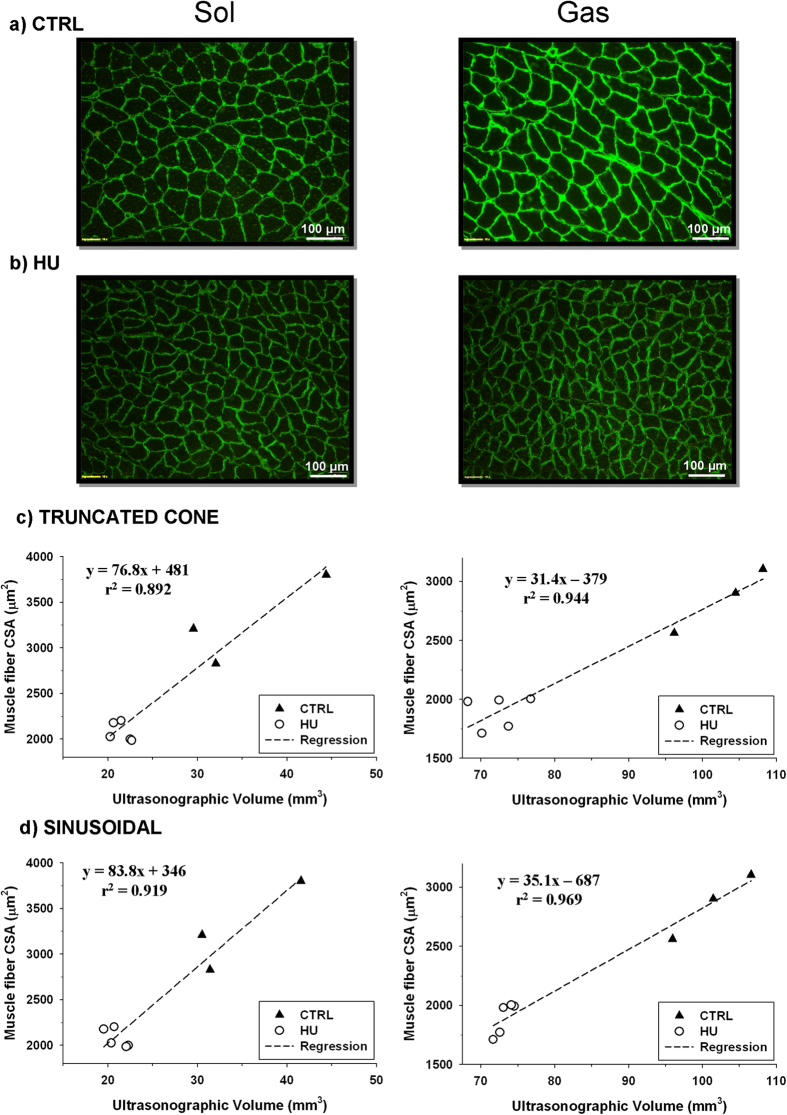
Comparaison between ultrasonographic volume and fiber cross-sectional area of Sol (left column) and Gas (right column) muscles. (**a**) Representative picture of muscle cryostat sections stained for laminin in control (CTRL) rats. (**b**) Representative picture of muscle cryostat sections stained for laminin in hindlimb-unloaded (HU) rats. (**c**) Linear correlation between ultrasonographic volumes determined with the truncated-cone formula and muscle fiber cross-sectional area in CTRL (n = 3) and HU (n = 5) rats. (**d**) Linear correlation between ultrasonographic volumes determined with the new sinusoidal method and muscle fiber cross-sectional area in CTRL (n = 3) and HU (n = 5) rats.

One of the most accurate method to evaluate muscle atrophy *ex-vivo* consists in the measure of muscle fiber cross-sectional area in muscle cryosections. We performed this analysis on laminin-stained cryosections of Sol and Gas muscles excised from 3 CTRL and 5 HU rats at D14 (Fig. 7a,b). In Sol muscle, the CSA was reduced from 3210 ± 283 in CTRL to 2026 ± 46 μm^2^ in HU (−36.9 ± 5.3%, P < 0.002 with unpaired Student’s *t*-test). In Gas muscle, the CSA reduction was from 2856 ± 158 in CTRL to 1892 ± 57 μm^2^ in HU (−33.8 ± 3.2%, P < 0.002 with unpaired Student’s *t*-test). These averaged values are closed to those of reduction of ultrasonographic volumes reported above (∼38% for Sol and ∼28% for Gas). Importantly, there was a strong linear correlation (r^2^ > 0.89) between the fiber CSA and the ultrasonographic volume of Sol or Gas muscles determined in the same animals, with either the truncated cone method (Fig. 7c) or the sinusoidal method (Fig. 7d).

This study shows, for the first time, the evaluation of skeletal muscle volume in rodents by using ultrasonography. We were able to monitor over time the Sol and Gas muscle atrophy in the same animals. Such a non-invasive technique represents a breakthrough in skeletal muscle research as it allows longitudinal *in vivo* studies, which would be especially helpful to measure muscle atrophy progression in rodent models of diseases (neuromuscular diseases, but also aging, cancer, virus infection, drug side effects, etc…) and to perform preclinical trials of candidate drugs and regenerative therapies. Although the ultrasound technique is reputedly limited to superficial muscles, thanks to the high frequency probe, we were able to measure the volume variation of the deep soleus and the more superficial gastrocnemius lateralis. Interestingly, the ultrasonography accessibility to both a slow-twitch, oxidative muscle (Soleus) and a fast-twitch, glycolytic muscle (Gastrocnemius), may be particularly relevant to study muscle phenotype-dependent atrophic processes. Besides its relative low cost, one of the advantage of the ultrasound technique with respect to other *in-vivo* imaging techniques, such as computerized tomography (CT) single photon emission computed tomography (SPECT), and positron emission tomography (PET), is the lack of ionizing radiations, which may be harmful especially during repetitive exams in longitudinal studies. Although it is noteworthy that magnetic resonance imaging (MRI) can provide reliable evaluation of skeletal muscle volumes, the MRI scanners are very expensive, may be affected by little movements of animals, and may require the use of contrast agents. Thus ultrasound sonography may appear as a very useful and accessible method to perform preclinical studies. Similarly, recent studies suggest that muscle ultrasonography may provide physiatrists with a practical and accurate tool for diagnosis of muscle atrophy in humans^20^,^21^.

We designed a novel method to evaluate the muscle volume. The novel sinusoidal method showed at least the same accurateness than the conventional truncated-cone method, as demonstrated by the linear correlation coefficients. In addition, the sinusoidal method allowed the final volume calculation by measuring only four parameters (A_1_, A_2_, d_1_, and d_2_), compared to fifty parameters for the truncated-cone method (Figs 2a, 3a and 5a). This aspect would imply an easier and faster analysis possibly with a lower margin of error.

In conclusion, our study provides a new method for the *in vivo* determination of rat muscle volume validating ultrasonography as a powerful approach for the evaluation of rat skeletal muscle atrophy. As already happened for cardiac physiology, ultrasound imaging has the potentiality to become a gold standard for the acquisition and measurement of skeletal muscle volume. Additional improvements may be obtained by extending this method to other skeletal muscles. The non-invasive ultrasound technique allows longitudinal studies *in vivo*, which may prove very useful for preclinical evaluation of skeletal muscle structural parameters in physiopathological conditions and after therapeutic intervention. Furthermore, the ultrasonographic volume evaluation could be a starting point for the non invasive assessment of functional parameters, such as the physiological cross sectional area and the power potential^3^. Last but not least, such technique would allow to decrease the number of animals needed to obtain statistically significant results.

## Materials and Methods

### HU experiments

The hindlimb unloading (HU) experiments were performed in accordance with the Italian Guidelines for the use of laboratory animals (d.Lgs 2014 n. 26), which conforms with the European Union Directive for the protection of experimental animals (2011/63/EU), and received approval from the Italian Ministero della Salute (D.M. n.133/2000-B). Adult male Wistar rats, weighting 295–360 g, (Charles River Lab., Calco, Italy) were randomly distributed into two groups: control (CTRL) and hindlimb-unloaded groups (HU). Two series of experiments were performed. The first experiment, including 6 CTRL and 6 HU rats, was performed to compare ultrasonographic volume to muscle weight-to-density ratio of the soleus muscle only. In the second experiment, including 3 CTRL and 5 HU rats, ultrasonographic volume, muscle weight-to-density ratio, and muscle fiber cross sectional area (CSA) were compared in both soleus and gastrocnemius muscles. Because the two experiments showed very similar results for Soleus muscle, data were pooled together. The HU rats were suspended individually in special cages for 14 days^10^. The rats were suspended by means of a shoelace linked at one extremity to the base of the tail by sticking plaster and at the other extremity to a trolley that can move on horizontal rails at the top of the cage^14^,^15^,^16^,^17^,^22^. The length of the lace was adjusted to allow the animals to move freely on their forelimbs, while the body was inclined at 30–40° from the horizontal plane. Control and suspended animals had food and water *ad libitum*. Animals were inspected each day for fine-tuning of suspension and control of animal health.

At day 0, 7 and 14 (D0, D7 and D14, respectively) the rats were weighted and ultrasound image acquisitions of the Sol and Gas muscles were performed. At D14, after ultrasonographic image acquisition, the rats were sacrificed by using an overdose of intraperitoneal injection of urethane and the Sol and Gas muscles from both legs were quickly removed and weighted. The Sol and Gas muscle volumes were calculated by dividing weight (g) by muscle density (0.00106 g/mm^3^), assuming that muscle density did not change between CTRL and HU rats^23^.

### Histological analysis

Gastrocnemius lateralis and soleus muscles, covered with tissue-tek O.C.T. (Bio-Optica), were frozen in isopentane cooled in liquid nitrogen in a slightly stretched position and stored at −80 °C. Serial cross sections (8-μm thick) were cut in a cryostat microtome set a −20 °C (HM525 NX, Thermo Scientific). To measure the cross-sectional area (CSA) of individual fibers, muscle sections were stained for laminin, a major component of the basal lamina, as previously described^24^. The sections were incubated with primary antibody against rabbit laminin (SIGMA Aldrich c.n. L9393) diluted 1:200 in PBS-gelatin for 1 h. After washing with PBS-gelatin, the sections were incubated with donkey anti rabbit IgG (Invitrogen c.n. A-21206) diluted 1:1000 in PBS-gelatin for 1 h, then washed with PBS-NaCl (300 mM). Sections were examined using Olympus CX41 microscope. Five digital photographs were taken of each muscle section and the CSA was measured as the internal laminin-unstained area by the ImageJ software (NIH, freeware imaging software). For each muscle sample, the number of counted fibers ranged between ∼350 and ∼900 fibers.

### Ultrasonography

Ultrasonography was performed using an UBM system (Vevo 2100; VisualSonics, Toronto, ON, Canada) (Supplementary Fig. 1a). For image acquisition, the rats were anaesthetized with isofluorane at a concentration of 1.5–2% and placed in the ventral decubitus position (Supplementary Fig. 1b). The hind limbs were shaved to avoid interference in the image and positioned strictly parallel to the rat body, with foot forming an angle of 90° with the hind limb. Image acquisitions were performed operating at a frequency of 40 MHz for B-Mode acquisition of Sol and Gas muscle images. The B-Mode images were generated with a 14 × 14 mm view field and at a frame of 15 Hz. Lateral and axial resolutions were 80 and 40 μm, respectively. For longitudinal image acquisition of Sol and Gas muscles, the probe was fixed parallel to the hindlimb (Supplementary Fig. 1b). Because of the unfeasibility to acquire an ultrasound image of the whole muscles in their length, a thin strip (2 mm) of micropore tape (3M, Bracknell, UK) was used to mark a virtual transversal division of the Sol and Gas muscles in distal and proximal parts with approximately similar lengths (Fig. 1a,b). Eye lubricant was placed on each eye to prevent drying of the area and a small amount of ECG gel was placed on the platform copper leads to allow ECG and respiratory recording. A rectal probe was used to monitor the temperature of the animal during the imaging session. An ultrasound gel was added between the animal skin and the probe, and a minimal pressure was applied to the muscle to minimize image distortion.

By the end of ultrasonography session, the rat was allowed to recover from anesthesia (usually 20 min) before to be re-suspended (D0 and D7) or immediately sacrificed by i.p. urethane overdose administration (D14).

### Utrasonographic evaluation of Soleus and Gastrocnemius muscle volume

The ultrasonographic evaluation of Sol and Gas muscle volume was performed by using the conventional truncated-cone method^12^, and a novel sinusoidal method.

The truncated-cone method consists in the calculation of the volumes of contiguous truncated cones drawn in the distal and proximal parts of the muscle (Fig. 2a,b). Each truncated cone volume is calculated from the equation,


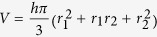


where r_1_ is the minor radius, r_2_ is the major radius, *h* is the height and π = 3.142 (Fig. 2c,d). The rat Sol and Gas muscles were subdivided in 8 contiguous truncated cones. The total volume of Sol and Gas muscles is calculated as the sum of all truncated cones volumes.

For the Sol muscle, the sinusoidal method was designed assuming that the muscle observed in the ultrasonographic window (sum of proximal and distal views) is spindle-shaped, with the fusiform profile resembling a sinusoidal function. The proximal and distal volumes can be approximated by the rotation around the tendon-to-tendon axis of a sine function, delimited by the measurements A_1_ and d_1_ or A_2_ and d_2_ (cyan lines showed in (Fig. 3a), and by a_1_ and a_2_ that are the sinusoidal arches (Fig. 3b).

Considering the proximal part, the volume is calculated considering the rotation around the hypotenuse *hyp* of an hypothetical right triangle with major cathetus *d*_*1*_ and minor cathetus (*A*_*1*_*/2)* (Fig. 3c). The *hyp* length can be calculated using the following formula:


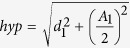


It is possible to approximate the length of *hyp* to the length of d_1_, which is easily measured on the acoustic window. The approximation introduced an error of only some undreadth of millimeter. Indeed, the *hyp* value calculated from the mean value of d_1_ and A_1_ at D0 (10.86 ± 0.12 (n = 12) and 1.94 ± 0.04 (n = 12), respectively) was 10.90 mm. thus the relative error between *hyp* and d_1_ calculated using the following formula [((*hyp* − d_1_)/d_1_) · 100] was only 0.4%. The same considerations apply to the distal part (Fig. 3c). Thus the Sol muscle volume can been calculated from the sum of the proximal and distal volumes calculated by the rotation around d_1_ and d_2_ of a sine function (Fig. 4a,b). We measured the A_1_ and A_2_ values by considering the greater thickness of the muscle and we calculated the A value as


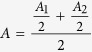


The sine function around the x axis with amplitude A and distances d_1_ and d_2_ returned the volume (Fig. 4c).

In order to apply the sine function, it is necessary to change the x variable (mm) in z variable (radians) using the following proportion:


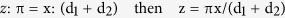


Replacing this new variable in the sine function, we obtained the following equation

f(x) = A sin z = A sin [π x/(d_1_ + d_2_)] (Fig. 4d).

This equation has been used to calculate the proximal and distal volume of soleus muscle, considering the solid of rotation applying the Guldino’s formula:


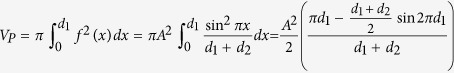


Similarly, the volume of distal part of the muscle is obtained from:


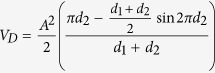


By summing V_p_ and V_d_, the whole Soleus muscle volume can be calculated as:


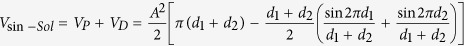


Finally, by using the Prosthaphaeresis formula, for which


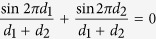


we concluded that the volume generated by the sine function around the x axis with amplitude A and distances d_1_ and d_2_ is:


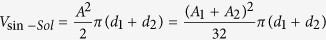


Since the gastrocnemius lateralis muscle is leaning on the soleus muscle, we assume that the lower profile of Gas follows a similar sinusoidal profile (Fig. 5a). Looking at the ultrasonographic window, also the upper profile of Gas muscle appears as a sinusoidal arc. The median line (d_1_), which is the line formed by all points equidistant from the arcs a_1_ and a_3_ for the proximal Gas (Fig. 5b), and the median line d_2_ equidistant from a_2_ and a_4_ arcs for the distal Gas, are symmetry lines between arcs a_1_, a_3_ and a_2_, a_4_. Above and below these symmetry lines, the profile of Gas muscle is sinusoidal. Therefore, d1 and d2 can be placed on an x axis to ​​obtain a geometrical model of the muscle volume (Fig. 4a,b). The whole volume is achieved by coupling proximal and distal parts (Fig. 4c). As for the soleus, considering the solid of rotation and applying the Guldino’s formula we get final formula to calculate the whole Gas volume:


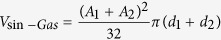


### Statistical analysis

Each point of the volume time-course relationship represents the mean ± standard error from n rats. Because no significant difference was found between the two hind limbs of each rat with either calculation method, the single rat value was calculated as the mean of the ultrasonographic volume of left and right soleus muscles. Statistical analysis was performed by Student’s paired *t* test within the groups or Student’s unpaired *t* test between the groups. Differences were considered significant for *p < *0.05 or less.

## Additional Information

**How to cite this article**: Mele, A. *et al*. *In vivo* longitudinal study of rodent skeletal muscle atrophy using ultrasonography. *Sci. Rep*. **6**, 20061; doi: 10.1038/srep20061 (2016).

## Supplementary Material

Supplementary Information
