# Supplementary material for: In vivo longitudinal study of rodent skeletal muscle atrophy using ultrasonography
Source: Sci Rep. 2016 Feb 1;6:20061. doi: 10.1038/srep20061 (PMC4735519; doi:10.1038/srep20061)
Supplement: Supplementary Information [file srep20061-s1.pdf]

In vivo longitudinal study of rodent skeletal muscle atrophy using ultrasonography

Antonietta Mele, Adriano Fonzino, Francesco Rana, Giulia Maria Camerino, Michela De Bellis, Elena Conte, Arcangela Giustino, Diana Conte Camerino, and Jean-François Desaphy

Jeanfrancois.desaphy@uniba.it

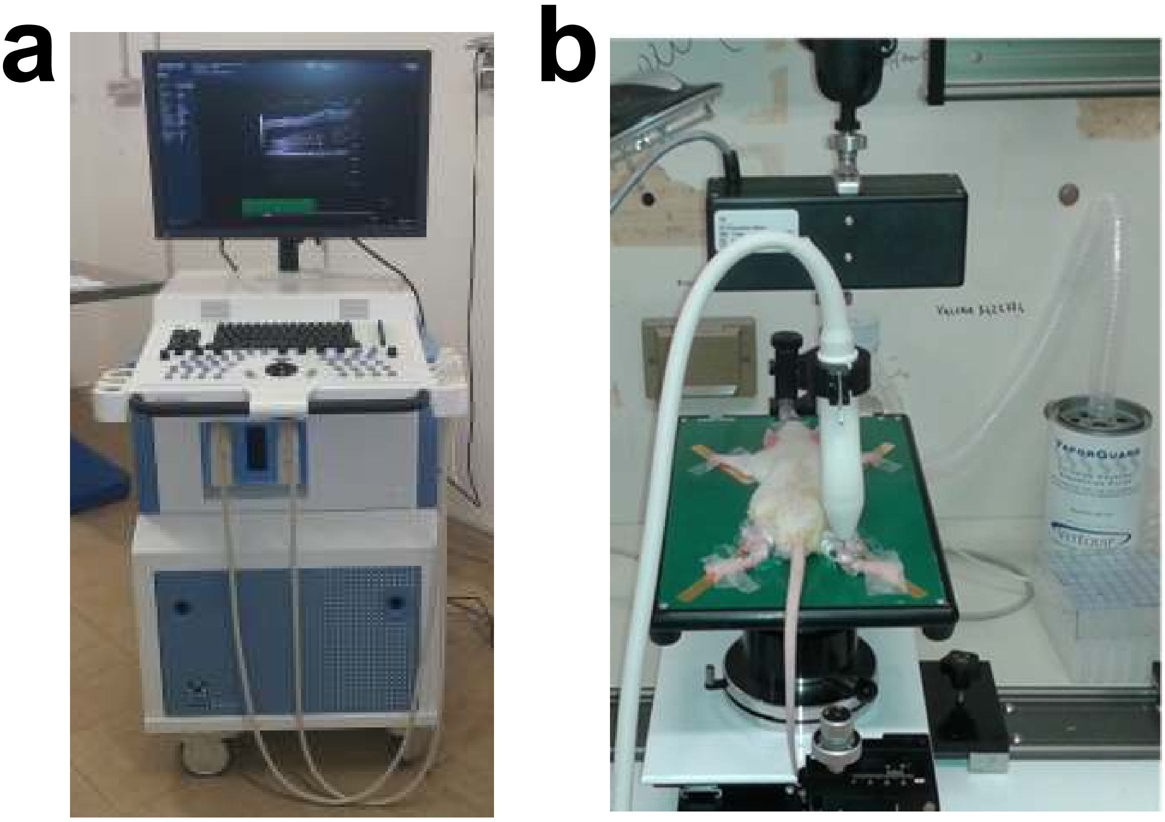

Supplemental Figure 1. (a) Image of ultrasound biomicroscopy (UBM) Vevo 2100 equipment **(b)** Image of rat's laying on ultrasound biomicroscopy (UBM) Vevo 2100 plate.
